# Full-wave acoustic and thermal modeling of transcranial ultrasound propagation and investigation of skull-induced aberration correction techniques: a feasibility study

**DOI:** 10.1186/s40349-015-0032-9

**Published:** 2015-07-31

**Authors:** Adamos Kyriakou, Esra Neufeld, Beat Werner, Gábor Székely, Niels Kuster

**Affiliations:** Foundation for Research on Information Technologies in Society (IT’IS), Zeughausstrasse 43, Zürich, 8004 Switzerland; Swiss Federal Institute of Technology (ETH) Zürich, Rämistrasse 101, Zürich, 8092 Switzerland; Center for MR-Research, University Children’s Hospital, Steinwiesstrasse 75, Zürich, 8032 Switzerland

**Keywords:** Acoustic modeling, Thermal modeling, Transcranial focused ultrasound, Focusing, Treatment envelope, Aberration correction, Treatment planning framework

## Abstract

**Background:**

Transcranial focused ultrasound (tcFUS) is an attractive noninvasive modality for neurosurgical interventions. The presence of the skull, however, compromises the efficiency of tcFUS therapy, as its heterogeneous nature and acoustic characteristics induce significant distortion of the acoustic energy deposition, focal shifts, and thermal gain decrease. Phased-array transducers allow for partial compensation of skull-induced aberrations by application of precalculated phase and amplitude corrections.

**Methods:**

An integrated numerical framework allowing for 3D full-wave, nonlinear acoustic and thermal simulations has been developed and applied to tcFUS. Simulations were performed to investigate the impact of skull aberrations, the possibility of extending the treatment envelope, and adverse secondary effects. The simulated setup comprised an idealized model of the ExAblate Neuro and a detailed MR-based anatomical head model. Four different approaches were employed to calculate aberration corrections (analytical calculation of the aberration corrections disregarding tissue heterogeneities; a semi-analytical ray-tracing approach compensating for the presence of the skull; two simulation-based time-reversal approaches with and without pressure amplitude corrections which account for the entire anatomy). These impact of these approaches on the pressure and temperature distributions were evaluated for 22 brain-targets

**Results:**

While (semi-)analytical approaches failed to induced high pressure or ablative temperatures in any but the targets in the close vicinity of the geometric focus, simulation-based approaches indicate the possibility of considerably extending the treatment envelope (including targets below the transducer level and locations several centimeters off the geometric focus), generation of sharper foci, and increased targeting accuracy. While the prediction of achievable aberration correction appears to be unaffected by the detailed bone-structure, proper consideration of inhomogeneity is required to predict the pressure distribution for given steering parameters

**Conclusions:**

Simulation-based approaches to calculate aberration corrections may aid in the extension of the tcFUS treatment envelope as well as predict and avoid secondary effects (standing waves, skull heating). Due to their superior performance, simulationbased techniques may prove invaluable in the amelioration of skull-induced aberration effects in tcFUS therapy. The next steps are to investigate shear-wave-induced effects in order to reliably exclude secondary hot-spots, and to develop comprehensive uncertainty assessment and validation procedures.

## Introduction

Transcranial focused ultrasound (tcFUS) under magnetic resonance imaging guidance (tcMRgFUS) has attracted the interest of the scientific and clinical community in recent years as a noninvasive and promising treatment modality. Initial clinical trials have indicated successful treatment of patients with brain tumors [1, 2], neuropathic pain [3, 4], and essential tremor [5–7]. In addition, clinical trials for the treatment of movement disorders, gliomas, obsessive compulsive disorder (OCD), and Parkinson’s disease are being set up in medical centers around the world [8]. Apart from the neurosurgical applications of FUS, this technology has been extensively evaluated for applications such as thrombolysis [9–13], blood-brain barrier (BBB) disruption for increased drug delivery [14–16], and even neuro-modulation [17–21].

Despite the substantial benefits of tcFUS when employed in the clinical setting, complications have been reported in human trials, typically in the form of unforeseen brain hemorrhaging [4, 22, 23]. The skull poses the largest barrier to the use of tcFUS; the combination of its complex heterogeneous nature and its significantly different acoustic properties compared to the soft tissue, i.e., twice the speed of sound and density and at least an order of magnitude higher absorption [24], can cause several undesirable effects. These include distortion of the acoustic energy deposition, shifting of the focus, and a significant decrease of the treatment’s focal gain, i.e., the ratio between the energy deposition at the focus and the energy deposition on the scalp and skull bone [25, 26].

The problems related to skull-induced aberrations have been partially resolved by employing large aperture, hemispherical transducer arrays that feature hundreds to more than 1000 elements, where each element is driven with an individual phase and amplitude [22, 27]. The large number of elements permits the acoustic energy to be distributed on the skull surface, thus diminishing the local deposition on the scalp and bone. In addition, the ability to drive the transducer elements individually with appropriately corrected phases and amplitudes allows for focal distortion effects to be partially compensated, but, especially at high acoustic frequencies, precise focusing is extremely important. A comprehensive review of analytical, numerical, and experimental skull-induced aberration correction techniques can be found in [28].

Moreover, the ability to predict and avoid unwanted secondary effects during the procedure, such as the development of standing pressure waves, especially in the case of low-frequency ultrasound or long sonications, or secondary hot-spots on the patient’s scalp and at bone-tissue and air-tissue interfaces, would be beneficial. Currently, this is feasible only through realistic acoustic and thermal numerical modeling of the entire procedure to derive the 3D pressure and temperature distributions.

The purpose of the current study is to introduce a novel integrated simulation framework that comprises both linear and nonlinear parallelized 3D full-wave acoustic solvers and powerful thermal solvers. These solvers have been tailored to simulate treatment setups involving heterogeneous anatomical models and are complemented by flexible geometric modeling, image segmentation, and post-processing tools that allow for rapid and precise acoustic and thermal modeling of FUS treatments. This framework was used to illustrate the feasibility of simulation-based optimization in a tcFUS therapy scenario. It was further used to investigate the impact of skull aberrations, compare different phase and amplitude correction approaches used to compensate for these aberrations, and achieve refocusing.

The simulated setup consists of an idealized model of the commercially available tcMRgFUS system ExAblate^®;^ Neuro (InSightec, Haifa, Israel) and the detailed anatomical head model of a 34-year-old, healthy male; 22 targets, in various locations in the brain of the head model, were defined and examined. Phase and amplitude corrections for each of these targets were calculated according to four different approaches, and the impacts on the acoustic focus and the induced temperature increase were investigated. In addition, the effect of temperature-dependent perfusion on the temperature distribution was also explored.

## Simulation framework

For the purposes of acoustic and thermal modeling of transcranial FUS propagation, an acoustic solver that allows for flexible 3D modeling of the simulation setup was developed and coupled to our existing thermal solver; the new solver has been integrated into our simulation platform SIM4LIFE (Zurich MedTech, Zürich, Switzerland) and optimized for anatomical model simulations, e.g., gridding and voxeling. In addition, an integrated medical image segmentation platform, iSEG (Zurich MedTech, Zürich, Switzerland), enabled the generation of patient-specific models and has been used to create high-quality reference anatomical surface models of healthy volunteers [29, 30]. The entire framework is supported by a versatile Python scripting interface, which allows the entire modeling, simulation, and post-processing procedure to be automated, and Visualization Toolkit (Kitware, New York, USA) based post-processing capabilities built into the platform to permit flexible visualization and evaluation of the results.

### Acoustic solver

An acoustic solver based on the finite-difference time-domain (FDTD) method [31–33] was developed to perform full-wave simulations.

Both linear and nonlinear variants, based on modified versions of the linear acoustic pressure wave equation (LAPWE) [34, 35] and the Westervelt-Lighthill equation (WLE) [36, 37], respectively, were developed. Both equations were extended with a density variation term to account for the change in density between neighboring voxels in the domain and to correctly capture the reflections at air-tissue and bone-tissue interfaces; FDTD stencils were tailored to nonuniform rectilinear grids to allow for flexible gridding of the computational domain. The LAPWE partial differential equation (PDE) is:

(1) $$ \rho\nabla\frac{1}{\rho}\nabla p-\frac{1}{c^{2}}\frac{\partial^{2} p}{\partial t^{2}} - \frac{\stackrel{\sim}{a}}{c^{2}}\frac{\partial p}{\partial t}=0   $$

and the WLE PDE is:

(2) $$ \rho\nabla\frac{1}{\rho}\nabla p-\frac{1}{c^{2}} \frac{\partial^{2} p}{\partial t^{2}}+\frac{\delta}{c^{4}} \frac{\partial^{3} p}{\partial t^{3}}+\frac{\beta}{2\rho c^{4}}\frac{\partial^{2} p^{2}}{\partial t^{2}}=0   $$

where *ρ* is the material density in kg m ^−3^, *p* is the acoustic pressure in Pa, *c* is the speed of sound in m s ^−1^, *a*∼ is calculated as $2a\sqrt {\frac {a^{2} c^{4}}{4\pi ^{2} f^{2}}+c^{2}}$ with *a* being the material attenuation coefficient in Np/m and *f* being the wave frequency in Hz. Parameter *δ* is the diffusivity of sound for a thermoviscous fluid and *β* is the nonlinearity coefficient. The nonlinearity coefficient *β* is related to the nonlinearity parameter of the fluid *B*/*A*, which is a pure number, by *β*=1+*B*/2*A*, while the diffusivity *δ* can be expressed as a function of the absorption coefficient of the fluid *α*, with $\delta =\frac {2\alpha c^{3}}{4\pi ^{2} f^{2}}$ [36, 37].

Numerical truncation of the computational domain was achieved by fitting the solvers with heterogeneous perfectly matched layer (PML) boundaries [38] that allow the simulated domain to be restricted to the volume of interest without introducing reflections. The formulation was based on the stretched-coordinate approach first proposed in [38] and subsequently derived for scalar wave equations in [39].

The two solver variants were parallelized for both multi-core systems using OpenMP and GPU devices using NVIDIA’s CUDA, resulting in a speedup factor of up to 45 times in the case of the latter, in order to allow the simulation of large computational domains in viable time frames.

In order to ensure the sound operation of the acoustic solvers developed within the presented framework, analytical, numerical, and experimental validation in water-tank setups was performed. For the sake of brevity, however, only the analytical and numerical validation of the solvers will be presented. Experimental validation of water-tank setup measurements employing focus-distorting obstacles, as well as validation against experiments involving ex vivo human calvaria, will be presented in an upcoming study.

### Thermal solver

A thermal solver tailored to biomedical applications has been previously developed, validated, and integrated into our simulation platform SIM4LIFE. The solver is based on a finite-difference implementation, with conformal corrections, of Pennes’ bioheat equation (BHE) [40]:

(3) $$ \rho C\frac{\partial T}{\partial t} = \nabla \cdot \left(k\nabla T\right) + \rho Q + \rho S - \rho_{b} c_{b} \rho \omega \left(T - T_{b}\right)   $$

where *ρ* is the material density, *C* is the specific heat capacity, *T* is the tissue temperature, *k* is the thermal conductivity, *Q* is the metabolic heat generation rate, *ω* is the perfusion rate, and *ρ*_*b*_, *c*_*b*_, and *T*_*b*_ are the density, specific heat capacity, and temperature, respectively, of the blood. The term *S* denotes the time-averaged rate of heat generation by relaxation absorption in a tissue of a continuous sound field. The formula for this term is [41]:

(4) $$ S = a\frac{p^{2}}{\rho c}   $$

By introducing this term into Eq. 3, it is possible to couple the two solvers and calculate the temperature induced in the tissue due to exposure to the acoustic fields. The thermal solver has the ability to account for thermoregulation and vascular shutdown and has been augmented with a wide range of perfusion models, including the discrete vasculature (DIVA) [42] and Weinbaum-Jiji (WJ) [43] models, support for MRI perfusion maps, etc., as well as Arrhenius tissue damage and thermal dose models [44], thus permitting realistic modeling and assessment of thermal effects in the body. In addition, the thermal solver allows boundary conditions to be applied to selected interfaces between different tissues or regions. Three types of boundary conditions can be employed:

- *Dirichlet*: A fixed temperature enforced at the interface (*T*=*T*_boundary_).
- *Neumann*: A fixed thermal energy flux enforced at the interface $\left (k\frac {dT}{dn} = F_{\text {boundary}}\right)$.
- *Mixed/Convenctive*: Energy flux that depends on the local surface temperature and equilibrates it to the specified environment temperature *T*_outside_ based on a heat transfer coefficient *h* in Wm ^−2^*K*^−1^, while in addition, a fixed heat flux can also be
  *F*_boundary_ added $\left (k\frac {dT}{dn} +h\left (T-T_{\text {outside}}\right) = F_{\text {boundary}}\right)$.

Further details on the implementation and validation of the thermal solver can be found in [45, 46].

## Acoustic solver validation

### Numerical validation

Numerical validation of the lossless and lossy LAPWE solvers was performed in simplified setups against the freely available software FOCUS [47–49]. FOCUS employs an entirely different model of acoustic wave propagation, namely the fast near-field method (FNM) [49–52], which is based on numerical approximations of analytical solutions and which has been extensively validated by its authors against the well-established software Field II [48].

Simplified transducers were initially simulated with the FNM module available in FOCUS. Subsequently, simulations of the same transducers were performed with the presented framework using the LAPWE model FDTD implementation. Three distinct transducers sonicating at 500kHz were simulated. These were a circular transducer with a radius of 10 mm, a rectangular transducer with dimensions of 20×20 mm, and a planar ring transducer with inner and outer radii of 7.5 and 10.0 mm, respectively. The transducers were embedded in an infinite homogeneous medium with acoustic properties akin to those of water, i.e., speed of sound *c* of 1500 m s ^−3^ and density *ρ* of 1000 kg m ^−3^. In order to separately validate the lossless and the lossy LAPWE solvers, two series of validations were performed with an attenuation coefficient *α* of 0.0 and 5.756 Np m ^−1^ [53], respectively.

The truncated computational domains had dimensions of 40×40×90 mm and were discretized with a 0.25-mm grid step, which amounts to *λ*/12, where *λ* is the acoustic wavelength for the given frequency and medium. In the case of the LAPWE solver, these domains were truncated with 16 layers of PML in order to inhibit the manifestation of spurious reflections at the domain boundaries.

While the FNM model directly calculates the steady-state pressure distribution, the acoustic solvers presented in this work are explicit, time-domain solvers using the FDTD method. Thus, to assess the necessary number of simulated periods required to achieve steady-state, multiple simulations over 50–90 periods were performed. It was ascertained that 60 periods were sufficient to achieve steady-state, as longer durations resulted in less than 0.1 *%* difference in terms of absolute pressure.

**Fig. 1:**
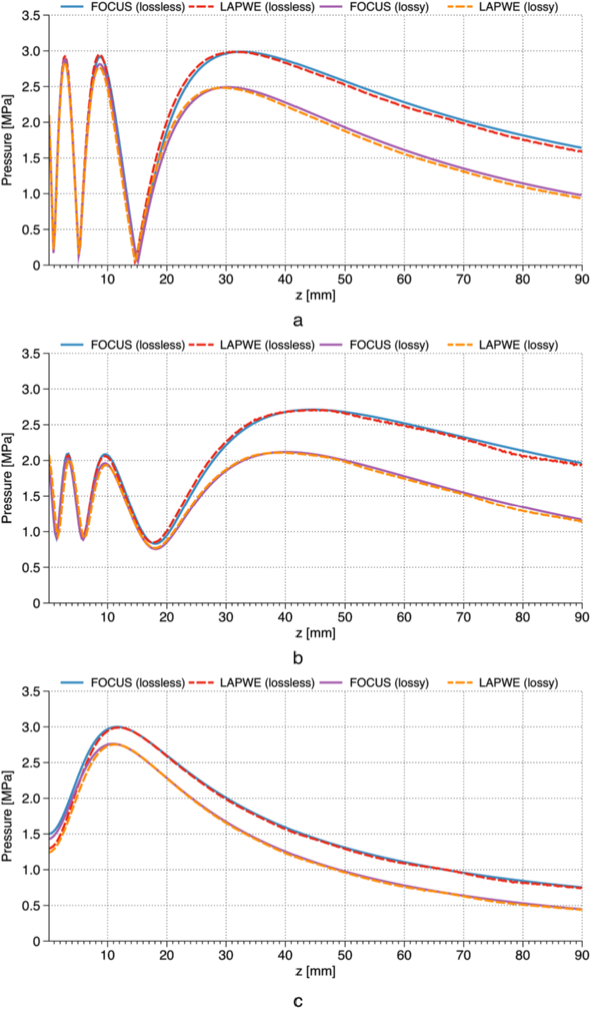
Validation of LAPWE against FOCUS. Absolute pressure line plots along the axis of propagation as calculated by both FOCUS and the LAPWE solver. The pressure comparisons within a lossless and a lossy medium can be seen for the cases of a circular (**a**), a rectangular (**b**), and a planar ring (**c**) transducers. Excellent agreement can be seen for all cases

The absolute pressure was plotted along the axis of propagation for both FOCUS and LAPWE in all six cases (see Fig. 1). Good agreement, with errors on the order of 1.9–3.5 % (see Table 1), was achieved between FOCUS and the LAPWE solver for all six comparison cases with only minor differences in the far-field regions, which are attributed to the cumulative phase dispersion errors inherent to the FDTD method [54]. In order to further quantify the agreement between the two pressure plots, the normalized standard deviation was calculated as the ratio of the *ℓ*^2^ norm of the pressure difference between the two lines and the *ℓ*^2^ norm of the FOCUS results:

(5) $$ \begin{aligned} \text{Normalized standard deviation \%}\\ = 100\frac{{\ell}^{2}\left(\left|p^{\text{LAPWE}}\right|-\left|p^{\text{FOCUS}}\right|\right)}{{\ell}^{2}\left(\left|p^{\text{FOCUS}}\right|\right)} \end{aligned}  $$

**Table 1:** The normalized standard deviation values between the FOCUS and the LAPWE solver presented in this work. Good agreement between the two solvers can be seen for all transducer types and media examined

Transducer	Normalized standard deviation	
	Lossless medium (%)	Lossy medium (%)
Circular	3.1	3.5
Rectangular	1.9	3.4
Ring	2.7	3.1

with *ℓ*^2^ norm being the square root of the sum of squares of all absolute pressure values for the respective line. These results can be seen in Table 1.

### Analytical validation

In order to validate the newly introduced density variation terms (see “Acoustic solver” section), which are not available in other numerical implementations, analytical validation based on the calculation of the reflection *R* and transmission *T* coefficients [55] at the interface between media with varying characteristic acoustic impedances *Z* was also performed. A 1-MHz acoustic plane wave was propagated through media with varying speeds of sound *c* and densities *ρ* at different incidence angles *θ*_*i*_. The reflection *R* and transmission *T* coefficients were calculated both analytically, through the characteristic acoustic impedances of the involved media and, numerically, through the pressure amplitudes of the incident, reflected, and transmitted waves at the interfaces of the different media based on the formulas below:

(6) $$\begin{array}{@{}rcl@{}} R = \frac{p_{r}}{p_{i}} = \frac{\left(Z_{2}\cos\theta_{i} - Z_{1}\cos\theta_{t}\right)}{\left(Z_{2}\cos\theta_{i} + Z_{1}\cos\theta_{t}\right)} &&\\ T = \frac{p_{t}}{p_{i}} = \frac{\left(2Z_{2}\cos\theta_{i}\right)}{\left(Z_{2}\cos\theta_{i} + Z_{1}\cos\theta_{t}\right)} \end{array} $$

where *p*_*i*_, *p*_*r*_, and *p*_*t*_ are the pressure amplitudes of the incident, reflected, and transmitted waves, respectively. The transmission angle *θ*_*t*_ of a plane wave propagating from a medium A to a medium B with incidence angle *θ*_*i*_ was calculated based on Snell’s law:

(7) $$ \frac{\sin\theta_{i}}{c_{A}} = \frac{\sin\theta_{t}}{c_{B}}  $$

where *c*_*A*_ and *c*_*B*_ are the speeds of sound in media A and B, respectively.

Nine such simulations were performed where the incidence of a plane wave from a medium with a speed of sound *c* of 1500 m s ^−1^ and density *ρ* of 1000 kg m ^−3^ on three different media at incidence angles of 0°, 15°, and 30° was modeled. Both the analytically (reference) and the numerically determined coefficients can be seen in Table 2. They are in excellent agreement within the numerical accuracy, thus verifying the correct implementation of the density variation and acoustic propagation term in the LAPWE solver.

**Table 2:** Analytical and numerical calculations of the reflection *R* and transmission *T* coefficients for the nine different cases. In the analytical case, the coefficients are calculated based on the acoustic impedance of the two media. The numerical calculations are based on the ratios between the pressure amplitudes of the reflected and transmitted waves to the amplitude of the incident wave

Setup						Analytical				Numerical				
Medium A			Medium B			Angles		Coefficients		*p* [MPa]			Coefficients	
*c* [m s ^−1^]	*ρ* [kg m ^−3^]	*Z*	*c* [m s ^−1^]	*ρ* [kg m ^−3^]	*Z*	*θ*_*i*_	*θ*_*t*_	*T*	*R*	*p*_*i*_	*p*_*t*_	*p*_*r*_	*T*	*R*
1500	1000	1.5	2000	1900	3.8	0	0.00	1.43	0.43	1.00	1.43	0.43	1.43	0.43
1500	1000	1.5	1400	600	0.84	0	0.00	0.72	−0.28	1.00	0.72	−0.28	0.72	−0.28
1500	1000	1.5	1200	600	0.72	0	0.00	0.65	−0.35	1.00	0.65	−0.35	0.65	−0.35
1500	1000	1.5	2000	1900	3.8	15	20.19	1.45	0.45	1.00	1.44	0.44	1.44	0.44
1500	1000	1.5	1400	600	0.84	15	13.98	0.72	−0.28	1.00	0.72	−0.28	0.72	−0.28
1500	1000	1.5	1200	600	0.72	15	11.95	0.64	−0.36	1.00	0.65	−0.35	0.65	−0.35
1500	1000	1.5	2000	1900	3.8	30	41.81	1.49	0.49	1.00	1.50	0.49	1.50	0.49
1500	1000	1.5	1400	600	0.84	30	27.82	0.71	−0.29	1.00	0.71	−0.28	0.71	−0.28
1500	1000	1.5	1200	600	0.72	30	23.58	0.62	−0.38	1.00	0.63	−0.37	0.63	−0.37

## Transcranial FUS simulations

The simulation framework described above was used to simulate a clinically relevant tcFUS scenario, in which a detailed anatomical head model segmented from MRI data was placed within the transducer cavity of the ExAblate^®;^ Neuro system. Four approaches, ranging from (semi-)analytical to simulation-based, were employed to calculate phase—and optionally amplitude—corrections of the skull-induced aberrations. The quality of the corrections was assessed both acoustically and thermally, and the different approaches were compared.

### Simulation setup

#### Head model

**Fig. 2:**
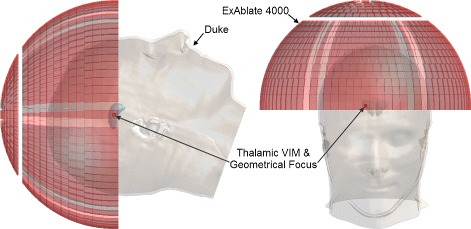
“Duke” anatomical head model and ExAblate^®;^ 4000 transducer array model. The “Duke” anatomical head model and the ExAblate^®;^ 4000 transducer array were used in this study. The positioning of the head model within the transducer as well as the location of the geometric focus in the right thalamic ventral intermediate (VIM) nucleus can be seen

**Fig. 3:**
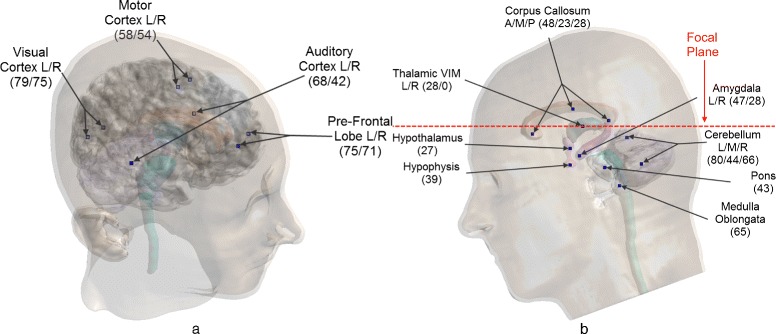
Sonication targets in the head model. The targets defined in the head model: **a** “cortex targets,” which all lie above the transducer’s focal plane, and **b** “structure targets,” where the *red dashed line* shows the transducer’s focal plane in relation to the targets, which heavily influences the achievable focusing. The initials *L*, *R*, *A*, *M*, and *P* stand for left, right, anterior, medial, and posterior, respectively, and the numbers in parentheses are the target distances in millimeter from the geometric focus of the transducer

The head model used in these simulations is an improved version of “Duke” of the “Virtual Population” collection of surface-based reference anatomical models segmented from MRI data of healthy volunteers [29, 30]. This improved version of the model, shown in Figs. 2 and 3, was segmented with iSEG (Zurich MedTech, Switzerland) from a re-sampled MRI dataset with a resolution of 0.5×0.5×0.5 mm, yielding over 50 individual tissues and anatomical structures in the head region alone. Additional details about the Virtual Population project can be found in [30].

#### Applicator model—ExAblate^®;^ Neuro

The applicator model used in these simulations was based on the ExAblate^®;^ Neuro (InSightec, Haifa, Israel), a tcMRgFUS system widely used in the FUS community, which is under evaluation for clinical safety and efficacy in functional neurosurgery, tumor ablation, and targeted drug delivery [1, 3–5, 8].

The ExAblate^®;^ Neuro consists of a 30 cm diameter hemispherical phased-array transducer with 1024 elements operating at either 230 or 650 kHz. This device is coupled with a 1024-channel amplifier, which allows phase and amplitude control of each individual transducer element in the phased array.

A model applicator (operating at a frequency of 230 kHz), shown in Fig. 2, was generated to mimic the actual applicator, with the same number of transducer elements in similar groupings. All elements were modeled identically, with a surface area of 1 cm ^2^.

#### Patient positioning and target definition

To replicate clinically relevant setups, the head model was placed so that the right thalamic ventral intermediate (VIM) nucleus was located at the geometric center of the transducer, i.e., where the acoustic waves of all the elements, driven in phase with no source of phase aberrations—e.g., the presence of the skull—would converge according to the arrangement of the elements in the array, as shown in Fig. 2.

To evaluate the different refocusing approaches that are described in the next section and the ability of each to focus the acoustic waves at a given location further away from the transducer’s geometric focus than is currently considered feasible, two types of targets, “structure targets” and “cortex targets”, were defined in the anatomical model. Structure targets, regions of the human brain that have been associated with different neuropathic conditions, are very desirable targets for tcFUS neurosurgery; 14 such targets were defined and are shown in Fig. 3b. In addition, eight cortex targets in different cortices of the brain were defined, as shown in Fig. 3a, which, because of close proximity to the skull, are thought by the FUS community to be untreatable.

### US simulations

#### Target focusing and aberration correction

To correct both amplitudes and phases for every element of the array, the LAPWE-based linear acoustic solver (see Eq. 1) was applied to each of the defined targets according to the following procedure:

- A point source, driven with an arbitrary amplitude, was placed at the intended target location.
- An inverse-propagation acoustic simulation of the entire head and applicator in which the waves from the point source were allowed to propagate for 1.0 ms, i.e., 230 periods at 230 kHz resulting in a propagation distance of ca. 1.5 m, was performed [56].
- During the simulation, the transducer elements were used as receivers to record the complex pressure values, i.e., amplitude and phase, at the surface center of every element.
- Four distinct focusing strategies were applied and evaluated as follows:
  1. *Distance-based phase corrections (DPC)*: Analytical phase corrections for every element were calculated based on the distance between each element’s surface center and the desired target, assuming that the transducer is in a homogeneous water medium, thus without wave distortion taken into account. The calculated distance-based phase corrections are applied to each element, the amplitudes of which are fixed to the appropriate pressure level for a given acoustic input power (see “Pressure levels” below).
  2. *Ray-tracing-based phase corrections (RTPC)*: As an extension of the DPC approach, a ray-tracing algorithm that takes the skull properties into account and allows calculation of improved effective distance-based phase corrections was devised. The algorithm calculates the skull entry/exit, i.e., intersection, points of the rays between each element’s surface center and the desired target and uses that information to calculate the thickness of the skull through which the waves from a given element propagate on the way to the target. Like in the DPC approach, the improved phase corrections and fixed pressure amplitudes are applied to each element.
  3. *Simulation-based phase corrections (SPC)*: The phases of the pressure phasors recorded during the inverse-propagation simulation are conjugated, and the amplitudes are fixed to the appropriate constant pressure level for a given acoustic input power.
  4. *Simulation-based phase and amplitude corrections (SPAC)*: The phases of the pressure phasors recorded during the inverse-propagation simulation are conjugated, and the recorded amplitudes, normalized to a given acoustic input power, are used (see “Amplitude normalization” below).
- Subsequently, for each of the four focusing strategies, the calculated phase—and in the case of the SPAC approach, amplitude—corrections were applied to their respective elements and a forward-propagation acoustic simulation was performed to investigate the acoustic and thermal impact of those corrections.

It should be mentioned that the SPC/SPAC approaches are based on the “Virtual Source” time-reversal approach more information on which can be found in [28, 57–59].

#### Gridding and voxeling

The simulation gridding was set up with at least 10 cells per minimum wavelength, which resulted in approximately 80×10^6^ voxels for each simulation.

#### Pressure levels

The definition of the actual pressure amplitude level(s) for the array elements for a given acoustic input power is pivotal to obtaining realistic pressure values at the focus location and to calculating the induced temperature increase. The following formula was used to calculate the average element surface pressure for a given acoustic input power:

(8) $$ p_{\text{element}} = \sqrt{\frac{P_{\text{total}} \cdot Z_{\text{water}}}{N_{\text{elements}} \cdot A_{\text{element}}}}  $$

where *p*_element_ is the pressure at the surface of an array element in Pa, *P*_total_ is the total acoustic input power in W, *Z*_water_ is the characteristic impedance of water in Rayl (or kg m ^−2^ s ^−1^ in SI units), *N*_elements_ is the number of active elements in the array, and *A*_element_ is the area of the element surface in m ^2^.

Assuming that water has a sound propagation speed of 1482.3 m s ^−1^ [25] and a density of 1000 kg m ^−3^, the resulting characteristic impedance *Z*_water_ is 1.4823×10^6^ kg m ^−1^ s ^−1^. Therefore, when all 1024 elements are active, sonicate at the same pressure, and have a surface area of *A*_element_= 1.0 cm ^2^, the pressure at the surface of every element for 1000 W acoustic input power is *p*_element,1000 W_≈ 120.31 kPa.

#### Amplitude normalization

In the SPC/SPAC approaches discussed prior, the complex pressure waves emanating from a point source with an arbitrary amplitude are captured, conjugated, and re-emitted in a subsequent simulation to achieve refocusing. While uniform pressure amplitudes are used across all elements in the SPC approach, in the case of the SPAC approach, the pressure amplitudes of these waves must be normalized. The factor *f* used for normalization of the captured complex pressure values is defined as:

(9) $$ f= \sqrt{\frac{N_{\text{elements}} \cdot p^{2}_{\text{element}}}{ \sum_{i~=~1}^{N_{\text{elements}}} p^{2}_{\text{captured}_{i}}}}  $$

where *N*_elements_ is the number of active elements in the array, *p*_element_ is the pressure amplitude calculated in the previous section, and $p_{\text {captured}_{i}}\phantom {\dot {i}\!}$ is the pressure recorded at the *i*th element of the array during the inverse-propagation simulation.

#### Acoustic tissue properties

Although most artificial materials have been acoustically characterized for use in transducer manufacture or nondestructive testing (NDT), an extensive literature search revealed no comprehensive studies on the acoustic properties of human tissue, apart from the properties of the bone which were first investigated by Fry [24]. While promising projects dealing with the measurement of such properties are underway [60], the only existing literature is generally decades old and comprises empirically measured properties with large discrepancies between different datasets. Such sources can be found in [25, 55, 61–65].

Consequently, most numerical studies on ultrasonic wave propagation involve simple modeling of a skull surrounded by water, with the argument that the ultrasonic tissue properties show little heterogeneity between different soft tissues.

In this study, however, accurate modeling of the entire head anatomy including all segmented tissues was necessary to allow for acoustic and, especially, thermal modeling of the procedure. To that end, the properties reported in [61] were used, while the grouping of soft tissue types based on physical composition was required to account for the lack of an extensive property database. The acoustic properties used in these simulations are summarized in Table 3. The material density *ρ* values were based on the IT’IS Foundation Tissue Properties Database [66]. Given that the anatomical model used was based on MRI data, it was not possible to acquire voxel-specific bone properties through Hounsfield units; thus, constant acoustic properties were assigned per tissue.

**Table 3:** The acoustic tissue properties used in this study where *c* is the speed of sound and *a* is the material attenuation coefficient [25, 61]

Tissue type	c [m s ^−1^]	a/f [Np m ^−1^ MHz ^−1^]
Air	343	0.04
Blood	1575	1.7
Bone	3183	164
Brain	1565	8.6
Eye (lens)	1647	9
Eye (aqueous humor)	1537	6
Eye (vitreous humor)	1532	5
Fat	1478	7
Muscle	1581	11
Skin	1720	19.7
Tendon	1750	43
Water	1482.3	0.025

### Thermal simulations

Following the acoustic simulations, the deposited acoustic energy was calculated for every voxel of the computational domain, using Eq. 4, and used as input in the thermal solver (see Eq. 3).

To realistically model the entire treatment setup, convective thermal boundary conditions were applied at the interfaces between tissues and the water-bolus surrounding the head as well as at air-tissue interfaces, both for the internal air in head cavities and the air surrounding the head. The water temperature was fixed to 16 °C [3, 4], and a heat-transfer coefficient *h* of 70 W m ^−2^ K ^−1^ was applied [45]. Similarly, both internal and external air were fixed to 25 °C with a heat-transfer coefficient *h* of 6 W m ^−2^ K ^−1^.

To properly account for the cooling effect of the water-bolus on the scalp, thermal simulations were performed for 30 min in the absence of sonication to allow the different tissues to reach thermal equilibrium, generally achieved after ca. 10 min. This was then followed by 20 s of sonication to calculate the temperature increase induced by the deposited acoustic energy during treatment. During these simulations, the effects of perfusion were taken into account, and the thermal properties for all tissues were based on [66].

These thermal simulations were repeated for every target in the head and aberration correction approach in order to calculate the induced temperature increase.

#### Vascular shutdown and temperature-dependent tissue perfusion

The above thermal simulations were performed with the assumption that thermal tissue properties are not temperature dependent. However, it is known that, during thermal ablation, the high temperatures induce vascular shutdown, thus eliminating perfusion in those locations and causing the temperature to increase more rapidly [67, 68]. To assess the importance of this effect, additional thermal simulations were performed for a few select targets where perfusion was assumed to start decreasing linearly when the tissue temperature exceeded 50 °C and cease entirely above 51 °C. Vascular dilation, which would cause an exponential increase in blood perfusion as a function of temperature, is not taken into account, as this effect manifests itself only after several minutes of exposure to increased local temperature [69–71].

## Results

The four aberration correction approaches described under the “Thermal simulations” section were applied to all targets shown in Fig. 3. The resulting pressure distributions were used to calculate the temperature increase as described under the “Thermal simulations” section.

Due to the target location diversity, the targets were loosely categorized as cortex targets, structure targets at or above the transducer’s focal plane, and structure targets below the focal plane.

To analyze the acoustic and thermal performance of the different approaches for each target in a consistent manner, an automatized local maxima and connected-component analysis was performed on all calculated 3D pressure and temperature distributions. Firstly, the distributions were filtered, and all local pressure/temperature maxima were identified. Subsequently, the local maximum that was nearest to the intended target was detected and assumed to denote the focal region/primary lesion. It should be noted that henceforth the term “lesion” will be used to refer to the region of the thermal hot-spot, even though in some cases, the intensity of the hot-spot might not be sufficient to create an actual lesion.

Once that maximum and the peak absolute pressure or temperature increase were identified, the simulated fields were thresholded at 50 *%* of the peak pressure or temperature level, and the different connected components were analyzed. This yielded the full-width half-maximum (FWHM) size of the focal region or thermal lesion along the *X*, *Y*, and *Z* axes, the distance between that region’s center and the intended target, as well as the volume of the region, calculated as the sum of the voxel volumes belonging to the particular component. The results of the analyses are summarized in Table 4 for the acoustic pressure distributions and in Table 5 for the temperature increase distributions.

**Table 4:** The results, mean (standard deviation), of the connected-component analysis on the absolute acoustic pressure distributions for the four focusing approaches for the different target categories

Quantity	Approach	ST above FP	ST below FP	CT	All targets
*D*[mm]	DPC	2.2 (0.6)	2.5 (1.0)	5.9 (6.4)	3.7 (4.3)
	RTPC	0.8 (0.1)	2.6 (1.7)	6.5 (8.3)	3.7 (5.6)
	SPC	0.5 (0.1)	0.7 (0.2)	0.7 (0.2)	0.6 (0.2)
	SPAC	0.6 (0.0)	0.6 (0.2)	0.6 (0.2)	0.6 (0.2)
*p*[MPa]	DPC	2.6 (0.5)	1.6 (0.5)	0.9 (0.3)	1.5 (0.7)
	RTPC	3.2 (0.3)	1.6 (0.6)	1.0 (0.3)	1.7 (0.9)
	SPC	4.6 (0.2)	3.2 (0.6)	2.5 (0.3)	3.2 (0.9)
	SPAC	5.1 (0.2)	3.8 (0.7)	3.2 (0.3)	3.8 (0.8)
*F*_shape_	DPC	1.8 (0.6)	1.7 (0.5)	4.2 (2.9)	2.6 (2.2)
	RTPC	2.1 (0.2)	1.9 (0.6)	1.0 (0.3)	1.7 (0.9)
	SPC	1.6 (0.2)	1.4 (0.3)	1.3 (0.1)	1.4 (0.2)
	SPAC	1.5 (0.1)	1.5 (0.4)	1.4 (0.1)	1.5 (0.3)
*V*[mm ^3^]	DPC	185.7 (52.4)	303.4 (386.4)	225.1 (256.6)	253.5 (310.3)
	RTPC	78.5 (2.8)	250.3 (543.8)	196.3 (238.1)	244.9 (407.4)
	SPC	51.6 (3.5)	50.3 (11.8)	38.3 (1.3)	46.2 (10.1)
	SPAC	50.8 (3.5)	51.2 (19.8)	48.1 (7.7)	50.0 (14.3)

*D* is the distance between the intended target and the nearest focal region, *p* is the peak absolute pressure in that region, *F*_shape_ is the shape-factor, i.e., the ratio of the maximum to minimum dimensions of the focal region, and *V* is the volume of that region. *ST* structure targets, *CT* cortex targets, *FP* focal plane

**Table 5:** The results, mean (standard deviation), of the connected-component analysis on the temperature increase distributions for the four focusing approaches for the different target categories

Quantity	Approach	ST above FP	ST below FP	CT	All targets
*D*[mm]	DPC	0.9 (0.3)	2.3 (2.3)	11.0 (10.6)	5.2 (7.9)
	RTPC	0.6 (0.2)	3.0 (2.7)	8.7 (9.5)	4.7 (6.8)
	SPC	0.3 (0.1)	0.8 (1.1)	0.5 (0.2)	0.6 (0.8)
	SPAC	0.3 (0.1)	0.5 (0.1)	0.5 (0.1)	0.5 (0.1)
*T*[°C]	DPC	17.9 (6.1)	6.2 (3.9)	1.7 (0.7)	6.7 (6.8)
	RTPC	23.2 (4.1)	6.5 (4.2)	1.7 (0.5)	7.8 (8.3)
	SPC	42.2 (3.5)	19.7 (6.5)	11.0 (2.0)	20.6 (11.9)
	SPAC	51.9 (4.2)	27.4 (8.6)	21.3 (4.3)	29.6 (12.7)
*V*[mm ^3^]	DPC	134.0 (47.4)	434.0 (594.9)	450.4 (544.2)	385.4 (532.1)
	RTPC	71.0 (4.7)	305.6 (386.2)	214.5 (176.6)	229.8 (294.0)
	SPC	51.5 (3.6)	62.6 (43.8)	38.5 (3.9)	51.8 (31.6)
	SPAC	51.3 (4.8)	52.0 (11.0)	66.9 (35.7)	57.3 (24.0)

*D* is the distance between the intended target and the nearest lesion, *T* is the peak temperature increase in that lesion, and *V* is the volume of that lesion. *ST* structure targets, *CT* cortex targets, *FP* focal plane

**Fig. 4:**
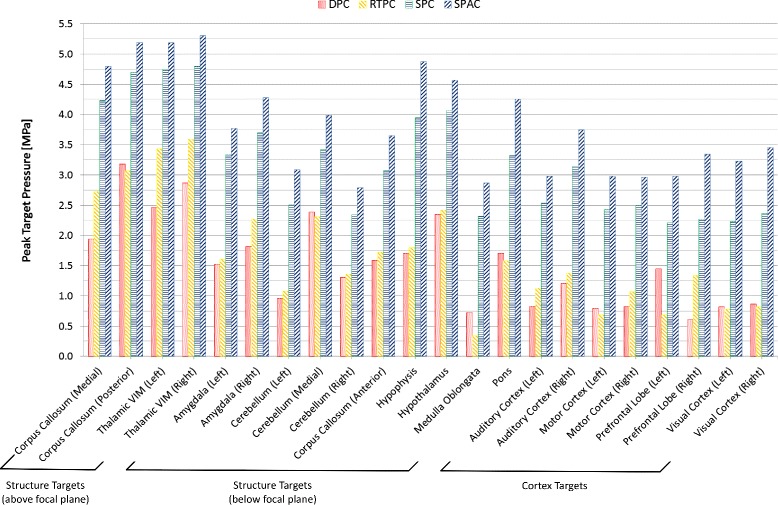
Peak target pressure for different targets and focusing approaches. The peak absolute pressure (in MPa) achieved near each of the defined targets for all focusing approaches. It can be clearly seen that the SPC and SPAC approaches yield far stronger foci than the DPC and RTPC approaches

**Fig. 5:**
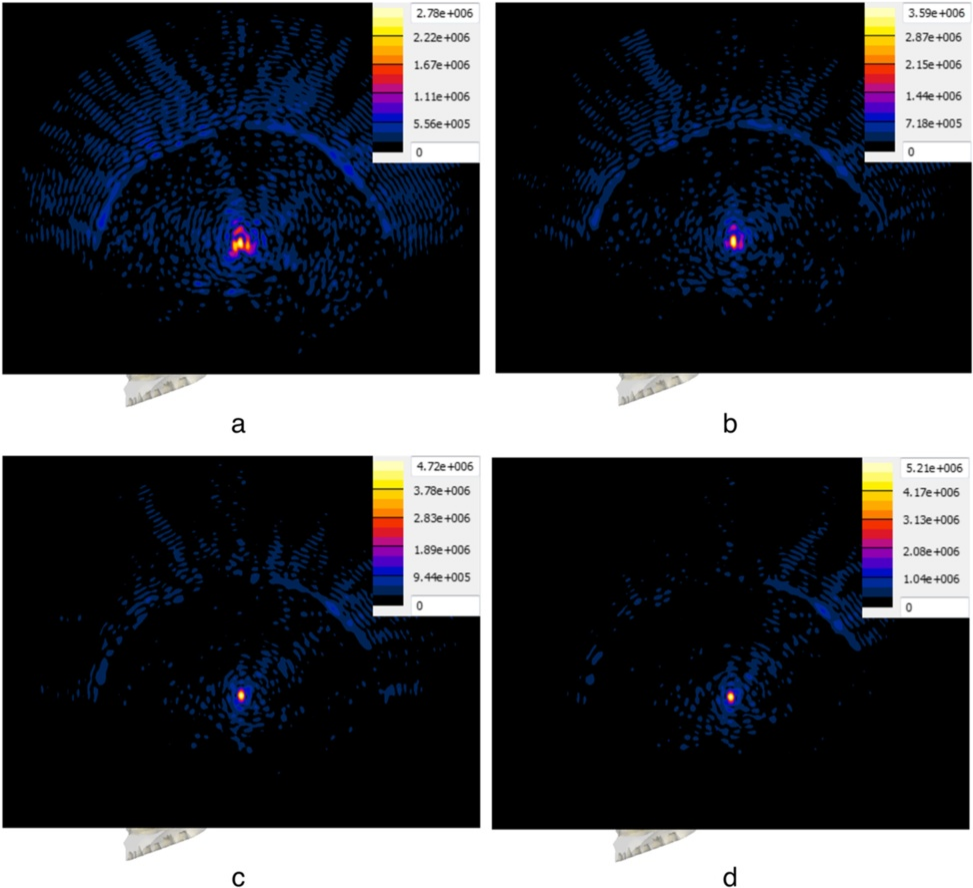
Pressure distribution comparison for different focusing approaches. Absolute pressure distribution in megapascal resulting from the use of the four approaches with the “Thalamic VIM (*right*)” target. Each distribution is plotted on the sagittal plane through the target, accompanied by the respective color map and scaled to the respective maximum absolute pressure. The distribution resulting from the DPC approach **a** shows a heavily distorted focal region and relatively low pressure amplitude at the target. Delineation of the focal region and amplitude improve slightly with the RTPC approach (**b**), while a significant improvement is seen in the case of the SPC (**c**) and SPAC (**d**) approaches

**Fig. 6:**
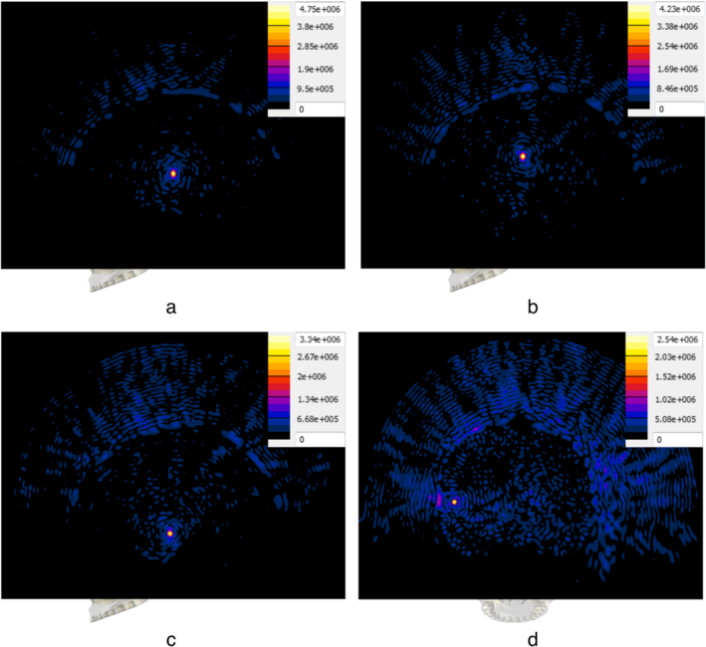
Pressure distribution comparison for four types of targets. Absolute pressure distribution in megapascal resulting from the use of the SPC approach with four selected targets. Each distribution is plotted on a plane through the target, accompanied by the respective color map and scaled to the respective maximum absolute pressure. Distributions **a**–**c** are plotted on the sagittal plane, while distribution **d** is plotted on the coronal plane. The two structure targets above the transducer’s focal plane, “Thalamic VIM (*left*)” (**a**) and “Corpus Callosum (*medial*)” (**b**), show sharply delineated focal regions with very high pressure amplitudes. The resulting distribution for “Amygdala (*left*)” (**c**), a structure target below the focal plane, shows successful refocusing but significantly lower pressure amplitude. In the case of the cortex target “Auditory Cortex (*left*)” (**d**), a focus is visible at the target location but significant energy deposition in the patient’s scalp and skull is observed

A plot of the peak absolute pressure in these detected foci for all targets and approaches is shown in Fig. 4. The absolute pressure distribution obtained with the four approaches, in the case of the “Thalamic VIM (right)” target—which coincides with the geometric focus of the transducer—can be seen in Fig. 5. In addition, the absolute pressure distributions resulting from the use of the SPC approach in the case of four selected targets can be seen in Fig. 6.

**Fig. 7:**
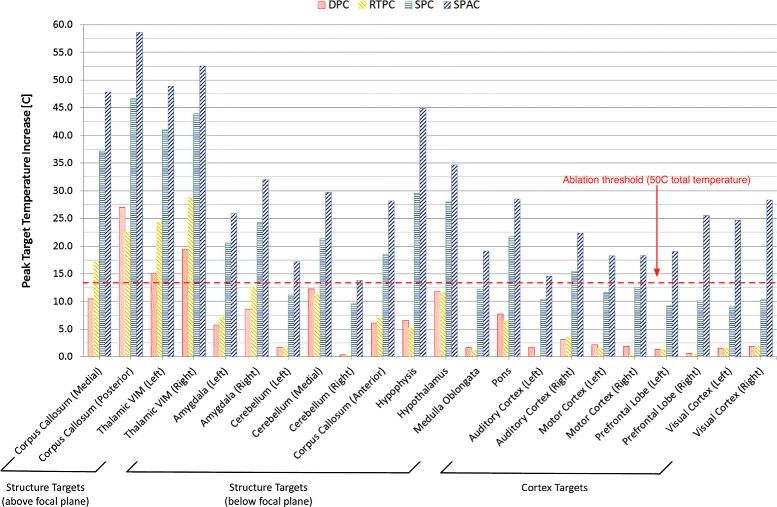
Peak target temperature increase for different targets and focusing approaches. The peak FUS-induced temperature increase, not absolute temperature, achieved with each of the defined targets for all focusing approaches after 20 s of sonication. The *red dashed line* shows the 50 °C threshold, assumed to be the ablation threshold for a base tissue temperature of 37 °C

A plot of the peak FUS-induced temperature increase achieved in each of the defined targets for all focusing approaches after 20 s of sonication is shown in Fig. 7.

## Discussion

### Acoustic distributions

As can be seen in Table 4, the acoustic pressure achieved in the focal regions nearest to the targets is lowest for the DPC approach—which neglects the impact of the skull—slightly higher for the RTPC approach (+10 *%* in average) and much higher for the SPC and SPAC approaches (+107 and +148 *%*, respectively). In terms of target location, structure targets above the transducer’s focal plane exhibited higher pressures than those below, while the achievable pressure in the cortex targets was the lowest.

The pressure level dependence on the focusing strategy can be mostly understood when the volume *V* of the different focal regions is considered. The phase corrections calculated through the DPC and RTPC approaches resulted in focal regions much larger, by a factor of 5.2 on average, than those obtained with simulation-based methods (see Fig. 5).

While the SPC and SPAC approaches yielded a nearly constant region size and shape for the different targets, focusing sharpness drastically decreased in the case of the DPC and RTPC approaches for structure targets below the transducer’s focal plane and even further for cortex targets, where dramatic size variations were observed. In general terms and regardless of the correction approach, focusing quality decreases as the distance between the intended target and the geometric focus of the array increases (see Fig. 6). As described by *F*_shape_, i.e., the ratio of the maximum to minimum dimensions of the focal region, in Table 4, the focal regions were more spherical for the simulation-based approaches than for the DPC and RTPC approaches, which yielded more elongated regions with substantial shape variations.

In terms of focal shift, precisions on the order of the discretization resolution were typically achieved with simulation-based approaches, as can be seen from *D* in Table 4, while the DPC and RTPC approaches showed an average shift of 3.7 mm, which was even more pronounced for cortex targets.

### Thermal distributions

When considering the temperature increase results after 20 s of sonication (see Fig. 7 and Table 5), the DPC and RTPC approaches achieved ablative temperatures only in the case of structure targets above the transducer’s focal plane. As observed with the acoustic results, temperature rise was highest for structure targets above the focal plane.

The SPAC was clearly superior to the other approaches and achieved ablative temperatures in all investigated targets. Utilization of the SPC approach attained ablation in all structure targets above the transducer’s focal plane and the majority of targets below. In the case of cortical targets, even though focusing was achievable with this approach, the limited number of elements that could contribute to the focusing (due to the absence of a line-of-sight between many of the elements and the intended target) resulted in high energy deposition on the skull and scalp, while the temperature rise at the targets showed inverse proportionality to the distance between them and the transducer’s geometric focus. Effective thermal treatment of those targets would require for the anatomical model to be repositioned so that the desired targets would lie above the focal plane.

The thermal lesion and acoustic focus volumes exhibited similar behaviors, with the simulation-based approaches being clearly superior to the DPC and RTPC, especially for the cortex and structure targets below the transducer line where these approaches were unable to produce sharply demarcated lesions.

### Skull heating

With the SPC approach, high acoustic energy deposition and subsequent thermal hotspots were observed near the skull surface for the majority of targets, which would result in significant heating of the scalp and skull. This phenomenon was partly alleviated for targets near the geometric focus with the use of RTPC, where improvements in thermal and focal gain were seen. This would suggest that, semi-analytical approaches, like the RTPC approach would be more appropriate for centralized targets, with longer sonication durations, or, for nonablative therapies.

In the case of the simulation-based approaches, these adverse effects were mostly observed for cortex targets and structure targets below the transducer level, where only a small number of elements could contribute to the focus, thus resulting in significant energy deposition on the scalp and skull bone (see Fig. 6). This trend was visible for both approaches, which leads us to conclude that these targets would benefit from further optimization of the steering parameters, e.g., deactivation of the nearby elements.

However, the pressure wave equation employed in this work does not capture shear-wave-related effects expected to occur near the skull, which is required to reliably predict the related secondary hot-spots. Therefore, further investigations are needed to extend the treatment envelope in clinical practice.

### Impact of vascular shutdown

As discussed under the “Vascular shutdown and temperature-dependent tissue perfusion” section, additional thermal simulations were performed where the impact of vascular shutdown was considered. The phase corrections of the SPC approach were used in vascular shutdown simulations for the “Thalamic VIM (left)” and “Thalamic VIM (right)” targets. The impact of vascular shutdown was visible but minimal. Thermal simulations where the perfusion was assumed to decrease linearly after the tissue temperature exceeded 50 °C and cease entirely after 51 °C showed a steeper temperature increase, but after 20 s of sonication, only 1 °C of the total additional temperature increase was observed in tissues where vascular shutdown occurred. Hence, this effect was considered to have negligible impact on the formation of thermal lesions.

### Impact of skull heterogeneity

The acoustic simulations performed in this study approximated the individual tissues as homogeneous, i.e., a single set of acoustic properties was assigned per tissue (see “Acoustic tissue properties” section). However, the particularly heterogeneous nature of the skull could have an impact on the degree of phase aberrations induced and on the quality of the subsequent compensation. To investigate this impact, additional acoustic simulations were performed utilizing the DPC and SPC approaches described in the “Thermal simulations” section in a setup comprising the applicator model discussed under the “Applicator model—ExAblate^®;^ Neuro” section and two models of a human skull based on a CT dataset. For these simulations, a sonication target coinciding with the geometrical focus of the applicator was defined.

The CT dataset of an ex vivo scan with a resolution of 0.48×0.48×2.5 mm was segmented based on Hounsfield units (HU) thresholding where HU values greater than 700 were considered to signify bone structures [72]. The HU values were then used to calculate the porosity and subsequently the density and speed-of-sound for each voxel in the skull, using the relationships from [73].

One of the generated models was fully homogeneous whereas the other one was partially heterogeneous and consisted 20 HU bins, within which voxels with similar HU values were grouped into a single region. This binning led to a maximum deviation of 2 *%* from the original HU values and was necessary for the current solver implementation which does not support individual tissue properties per voxel.

The results obtained are within range of the values for structure targets (ST) above the focal plane in Table 4. Comparing the pressure distribution in the homogeneous and the inhomogeneous skull model shows that the SPC approach produces identical shifts (ca. 0.5 mm) and focal region volumes (ca. 50.0 mm ^3^), while considering heterogeneity reduces the peak pressure by 25 *%*. A similar reduction of 35 *%* in peak absolute pressure amplitude was also observed with the DPC approach. In addition, the DPC approach in the case of the heterogeneous skull exhibited both more pronounced focal region shifts (factor of 3) and defocusing (33 *%* volume decrease) when compared to the homogeneous case.

These results from a single case indicate that the prediction of the achievable SPC-based focusing (shift and volume) is unaffected by skull inhomogeneity, while prediction of aberration effects could be overestimated when employing the DPC approach and using a homogeneous model. The assumption of image data-based skull property distributions results in lowered predicted peak pressure, possibly due to the increased reflections and scattering at the skull.

## Conclusions

A well-known limitation of tcFUS therapy is the skull-induced aberrations, which can induce focal shift and distortion as well as significant energy deposition on the patient’s skull and scalp, resulting in a significant decrease in the treatment’s focal and thermal gain. A numerical feasibility study was performed here to investigate the efficacy of four compensation techniques, ranging from (semi-)analytical to simulation-based, that aim to provide phase—and optionally amplitude—corrections to achieve refocusing, counter the aforementioned effects, and increase the treatment envelope of tcFUS therapy. To that end, an extensive, newly developed and validated simulation framework that allows for 3D full-wave, linear and nonlinear acoustic, and thermal simulations in large and complex clinically relevant setups, was utilized to perform simulations of a detailed anatomical head model sonicated with a model of the ExAblate^®;^ Neuro applicator. The acoustic and thermal results of these correction approaches were ascertained for 22 distinct targets in various locations of the brain. Good overall agreement in focal pressure and temperature can be seen between this and similar, recently published, numerical studies [74].

Evaluation of the acoustic pressure, location, and size of the focal regions as well as the FUS-induced temperature increase and lesion volume/size suggests that simulation-based approaches provide far superior corrections than the analytical and (semi-)analytical ones. While the latter could be employed in thermal interventions for targets in the vicinity of the transducer’s geometric focus, their efficiency decreased dramatically when targeting more remote brain regions. Simulation-based approaches, on the other hand, appear to yield sharply demarcated focal regions and lesions at targets multiple centimeter from the geometric focus, as well as the ability to better utilize and control large transducer arrays. Employment of modern but affordable computer hardware, combined with state-of-the-art high-performance computing techniques such as those described for the numerical framework presented here, enables realistic acoustic and thermal simulations in complicated setups to be performed within minutes. Due to their increased refocusing efficiency and their ability to predict the acoustic and thermal effects of FUS therapies, if extensively validated, simulation-based correction approaches may eventually replace their analytical counterparts.

In future work, additional experimental validation of the presented aberration correction approaches should be carried out against measurements of ex vivo human calvaria. Furthermore, the impact of bone density heterogeneity on the quality of aberration correction should be further investigated through employing MR and CT image data, the impact of shear-wave-induced effects must be considered to reliably predict and exclude secondary hot-spots, and the analysis needs to be extended to higher frequencies, e.g., the 650-kHz system. It is necessary to develop a comprehensive uncertainty assessment and validation procedure to enable these techniques to be used in treatment planning.
